# Biological databases in the age of generative artificial intelligence

**DOI:** 10.1093/bioadv/vbaf044

**Published:** 2025-03-20

**Authors:** Mihai Pop, Teresa K Attwood, Judith A Blake, Philip E Bourne, Ana Conesa, Terry Gaasterland, Lawrence Hunter, Carl Kingsford, Oliver Kohlbacher, Thomas Lengauer, Scott Markel, Yves Moreau, William S Noble, Christine Orengo, B F Francis Ouellette, Laxmi Parida, Natasa Przulj, Teresa M Przytycka, Shoba Ranganathan, Russell Schwartz, Alfonso Valencia, Tandy Warnow

**Affiliations:** Center for Bioinformatics and Computational Biology, University of Maryland, College Park, MD 20742, United States; Department of Computer Science, The University of Manchester, Manchester M13 9PL, United Kingdom; The Jackson Laboratory, Bar Harbor, ME 04609, United States; School of Data Science, The University of Virginia, Charlotesville, VA 22904, United States; Institute for Integrative Systems Biology, Spanish National Research Council, Paterna 46980, Spain; Bioinformatics & Systems Biology Graduate Program, La Jolla, CA 92093, United States; Department of Pediatrics, University of Chicago, Chicago, IL 60637, United States; Ray and Stephanie Lane Computational Biology Department, School of Computer Science, Carnegie Mellon University, Pittsburgh, PA 15213, United States; Institute for Bioinformatics and Medical Informatics, University of Tübingen, Tübingen 72076, Germany; Max Planck Institute for Informatics and Saarland Informatics Campus, Saarbrücken 66123, Germany; Dassault Systèmes BIOVIA, San Diego, CA 92121, United States; Elektrotechniek ESAT-STADIUS, University of Leuven, Leuven 3000, Belgium; Department of Genome Sciences, University of Washington, Seattle, WA 98195, United States; Department of Structural and Molecular Biology, University College London, London WC1E 6BT, United Kingdom; Montreal, QC H2T 2W5, Canada; IBM T J Watson Research, Yorktown Heights, NY 10598, United States; Computational Biology Department, Mohamed bin Zayed University of Artificial Intelligence, Abu Dhabi SE45 05, United Arab Emirates; Barcelona Supercomputing Center, Barcelona 08034, Spain; Institución Catalana de Investigación y Estudios Avanzados (ICREA), Barcelona 08010, Spain; Department of Computer Science, University College London, London WC1E 6EA, United Kingdom; Computational Biology Branch, Division of Intramural Research, National Library of Medicine, Bethesda, MD 20894, United States; Department of Chemistry and Biomolecular Sciences, Macquarie University, Sydney, NSW 2109, Australia; Ray and Stephanie Lane Computational Biology Department, School of Computer Science, Carnegie Mellon University, Pittsburgh, PA 15213, United States; Department of Biological Sciences, Carnegie Mellon University, Pittsburgh, PA 15213, United States; Barcelona Supercomputing Center, Barcelona 08034, Spain; Institución Catalana de Investigación y Estudios Avanzados (ICREA), Barcelona 08010, Spain; School of Computing and Data Science, University of Illinois Urbana-Champaign, Urbana, IL 61801, United States

## Abstract

**Summary:**

Modern biological research critically depends on public databases. The introduction and propagation of errors within and across databases can lead to wasted resources as scientists are led astray by bad data or have to conduct expensive validation experiments. The emergence of generative artificial intelligence systems threatens to compound this problem owing to the ease with which massive volumes of synthetic data can be generated. We provide an overview of several key issues that occur within the biological data ecosystem and make several recommendations aimed at reducing data errors and their propagation. We specifically highlight the critical importance of improved educational programs aimed at biologists and life scientists that emphasize best practices in data engineering. We also argue for increased theoretical and empirical research on data provenance, error propagation, and on understanding the impact of errors on analytic pipelines. Furthermore, we recommend enhanced funding for the stewardship and maintenance of public biological databases.

**Availability and implementation:**

Not applicable.

## 1 Introduction

Computational inference has been an integral part of the computational biology data ecosystem since the early days of the field. Data deposited in public databases feed inference algorithms that perform many useful tasks, such as identifying features of genomic sequences or biomedical images, assigning taxonomic or functional labels to biological sequences, or predicting the 3D structure of proteins. At the same time, the data stored in biological databases are frequently generated by inference algorithms, raising the question of whether this self-feeding loop may affect the quality of biological data sets or of the biological insights derived from them. This is not a new concern (Devos and Valencia 2001) and is prominently highlighted by a quote from Doerks and colleagues in 1998: “While [software] robots are the only solution to cope with the flood of data, they are also dangerous because they can currently introduce and propagate mis-annotations” (Doerks *et al.* 1998). Arguably, in hindsight, this concern may seem overstated, given that the data revolution has led to many biological discoveries that have substantially advanced biomedical knowledge. While errors are undoubtedly present in databases, their ultimate impact on computational biology appears to have been limited, either because errors cancel each other out, because they are washed out by the sheer volume of data available, or because their effects are mitigated by careful analyses and follow-up experiments (e.g. Rembeza and Engqvist 2021). However, such validation is expensive and diverts part of the scarce resources available for biological research. Furthermore, the impact of error propagation in databases may not be fully appreciated because there have been limited systematic attempts to quantify database errors.

We do not want to diminish the substantial effort that is being undertaken by database maintainers to ensure the quality of the data stored in biological databases. Both manual and automatic quality assessment and data curation tools ensure that publicly available data sets and knowledge are generally reliable resources for the scientific community. Furthermore, multiple communities of stakeholders are affected by and actively discussing issues such as those we outline below. We argue, however, that the recent advances in generative artificial intelligence (AI) warrant a new exploration of the interaction between biological data and the computational inference tools used to analyse and generate data sets. As generative AI and computational inference become even more widely used in computational biology research, and as the volume of synthetic data sets being generated is rapidly increasing—e.g. the Research Collaboratory for Structural Bioinformatics Protein Data Bank now contains five times as many structure predictions made by AlphaFold as experimentally-supported structures (Burley *et al.* 2023)—it is important to develop a better understanding of error propagation and to establish evidence-based best practices to reduce the chance that computationally inferred data are misused or inadvertently used in ways that affect subsequent conclusions or that reduce the power of developed models.

This perspective is not intended as a broad exploration of all the ways in which AI is or may be used in biological data curation and annotation. Rather, we focus specifically on concerns that lie at the interface of biological data and computational inference with the goal of inspiring increased research and educational activities in this space. We make recommendations that will apply broadly across the diversity of data types and computational techniques represented in modern biology research.

## 2 Biological databases

Biological databases (generally defined) serve two important roles. They can be archives of biological data and their interpretation as understood when the data were deposited, or repositories of data and the current knowledge about the data. To distinguish these roles throughout the article, we will refer to repositories that have a primarily archival role as ‘databases’ [e.g. GenBank (Bilofsky *et al.* 1986)], and to systems that curate, organize, and summarize current biological knowledge as ‘knowledgebases’ [e.g. Kbase (Arkin *et al.* 2018)]. In databases, error detection and curation take place only at submission, while knowledgebases require constant stewardship to ensure the data reflect current biological knowledge. While we make this distinction between databases and knowledgebases, many biological repositories fall somewhere in the middle of the continuum between a purely archival role and a constantly updated repository of current knowledge.

## 3 Data in biological databases

Biological databases typically contain data derived from experiments. Some examples include DNA sequences (generated from biological molecules by sequencing instruments), measurements of the abundance of molecules (e.g. gene expression levels estimated by microarrays or sequencing, peptide abundances estimated through mass spectrometry), 3D structure information (estimated through X-ray crystallography, NMR, or cryo-EM), or imaging data (e.g. from patient scans, X-rays, or microscopy). Even when the primary source of the data is an experiment, computational tools are frequently used to process and to convert the experimental data into the format that is ultimately stored in the database (e.g. sequence assembly). Nonetheless, the resulting data are commonly viewed as “experimentally derived.” Scientists assign them a higher value because they capture properties of the natural world even though experimental and analytic errors occur. Computational annotation tools, or, in some cases, human curators, are then used to assign labels to the primary data, such as taxonomic or functional labels. The annotations can be either experimentally derived or determined through rule-based inferences. For example, annotations are frequently inferred by similarity, transferring annotations from a better-studied sequence to one putatively ancestrally related to it. Scientists who rely on such annotations for hypothesis generation and expert knowledge must know their provenance and the pitfalls and biases inherent in making inferential assertions (Gaudet and Dessimoz 2017). While most databases and knowledgebases provide a wealth of information about the provenance of data and associated annotations, many users either do not understand or ignore this information, particularly when the data are used as input to large-scale computational analyses. Further, computational biologists generally understand annotations to represent interpretations of biological observations when the annotation was made. However, historical labels may be interpreted as current by individuals who need more training in assessing the relevance to current research of different data sources.

It is generally assumed that the primary data stored in databases are derived from experimental sources. In contrast, the annotations assigned to these data may or may not have an experimental origin. There are, however, instances where the primary data themselves may be computationally inferred—a common example is imputation, where a missing data element is replaced with an artificial data point inferred from contextual information. Imputation reduces the need for costly wet-lab genomic experimentation by supplementing limited experimental data with computationally imputed data. Recently, advances in protein structure prediction using neural networks (e.g. AlphaFold) have led to the creation of entire databases of computationally inferred protein structures (Varadi *et al.* 2022). AI technologies are also used in synthetic biology to generate new chemical compounds (Meier *et al.* 2020) or proteins (Ferruz *et al.* 2022, Madani *et al.* 2023). Computational generation of data is also used to preserve patient privacy in certain medical applications—e.g. a hospital may use data from its patients to generate a collection of virtual patients that capture key features of the real patients without revealing any personally-identifiable information, thus allowing the hospital to share the medical data with external researchers (Gonzales *et al.* 2023).

Generative AI has made it easier to generate large volumes of data that are practically indistinguishable from those we traditionally expected to be experimentally derived. At the extreme, digital twins—computational models that emulate the behavior of a biological process, cell, organ system, or organism—can allow researchers to conduct virtual experiments, resulting in data and findings that mimic those from a real experiment. It is critical that such computationally generated data items are clearly labeled so that they can be appropriately interpreted by both data users and analytic pipelines. A good example is the prominent way in which LLM-generated summaries for protein families, particularly for PANTHER entries (Mi *et al.* 2021), are flagged in the InterPro database (Blum *et al.* 2025).

## 4 Errors in public databases

Biological experiments are imperfect and can generate erroneous data, and further errors can be introduced in databases by the computational tools used to interpret experimental data. Such errors can affect the accuracy of the computational tools that rely on the information represented in databases, consistent with the well-known adage “garbage in, garbage out.” Database errors are unavoidable (Devos and Valencia 2000) and the extent of error in current biological databases is poorly understood; however, several studies have revealed systematic errors that impact biological analyses. In Percudani *et al.* (2013), the authors highlighted a set of enzymes that have had their function repeatedly mis-annotated in databases and publications owing to an incorrect interpretation of the function assigned to a homologous enzyme. The initial mis-annotation had been made in the 1990s, yet its consequence persisted for more than 20 years, raising questions about the time horizon of the presumed self-correcting nature of science. In Schnoes *et al.* (2009), the authors focused on 37 enzyme families for which substantial experimental evidence was available. They revealed extensive mis-annotation of these families across multiple databases, affecting up to 80% of some enzyme superfamilies. A similar estimate of the error in enzyme annotation (78%) was revealed by a careful experimental and computational exploration of a specific enzyme function (EC 1.1.3.15: *S*-2-hydroxyacid oxidases) in the BRENDA database (Rembeza and Engqvist 2021). Another example, highlighted in Attwood *et al.* (2009), shows a mis-annotation of a G protein-coupled receptor initially caused by the incorrect interpretation of experimental data. This mis-annotation is nonetheless propagated through databases. Several studies highlighted the extent of contamination in genomic databases, including bacterial contamination of eukaryotic genomic data and vice versa (Langdon 2014, Steinegger and Salzberg 2020). Such contamination can hamper a range of analyses, such as diagnostic tests or genetic variant analyses, and has possibly resulted in the creation of spurious protein families (Breitwieser *et al.* 2019).

Today, attempts to ensure data quality are either based on quality checks applied to data as they are deposited into databases or are part of regular maintenance or updates of biological knowledgebases. As the volume of data sets being deposited is rapidly increasing, manual validation of data is becoming impractical or impossible, and even computational validation is often restricted to analyses that can be performed rapidly and with limited computational resources. Furthermore, no computational tools are currently available that can identify computationally-generated data. Thus, the integrity of databases relies on the data submitters to correctly identify the provenance and quality of the data being deposited.

## 5 Error propagation in databases

Database errors can be propagated and amplified when annotation tools assign labels to database entries on the basis of training data that themselves were computationally annotated (Valencia 2005). This situation, usually referred to as “transitive annotation,” is likely to be common, in our opinion, as most labels in databases are inferred computationally rather than derived from experimental evidence. For example, Mahlich *et al.* (2018) reported that, in 2017, only ∼7000 out of 214 000 proteins with unique enzyme commission (EC) number annotations in the UniProt database were annotated on the basis of experimental evidence, with the rest derived through the application of computational tools. While it is well accepted that the transitive annotation process can induce errors, it has yet to be extensively studied, theoretically or empirically, in computational biology beyond some initial studies in limited settings (Muralidharan *et al.* 2023). The issues arising from training data being generated by AI models have started to be explored in the broader machine learning community, with initial results described in the context of natural language and image analysis. These results suggest that the repeated training of machine learning models on data that were themselves generated by the models can lead to the erroneous behavior of classification algorithms (Martínez *et al.* 2024), termed “model collapse” (Shumailov *et al.* 2024) or “model autophagy disorder” (Alemohammad *et al.* 2024). Such behavior is, in part, due to a homogenization of the model as training iterations reinforce each other. The extent to which such behavior occurs for the classification tasks common in computational biology, particularly beyond the scope of deep-learning classifiers, remains an unexplored but essential area of research.

A further challenge is posed by the fact that biological databases are frequently dependent on each other. The data and annotations in one database may be derived from data in one or more different databases which themselves may be derived from different sources. We believe that errors may, thus, propagate within the sequence database network (Goudey *et al.* 2022) or emerge owing to subtle inconsistencies between the connected databases. A better understanding of the structure of the database network can provide opportunities for detecting and even correcting data errors (Goudey *et al.* 2022). However, building the database network itself will require accurate and appropriately detailed provenance information.

## 6 Provenance and usage

To track errors within and across databases and to avoid training a model on data that were generated by one or more algorithms, it is necessary to track the provenance of each data item in a database. Many databases record such information [e.g. “evidence” tags in UniProt, “experimental/inference” codes in GenBank (Sayers *et al.* 2023), or commonly used cross-reference links to other databases], although it is unclear whether this information is routinely used when building training data sets for inference tools. Furthermore, it is the responsibility of the data submitters to provide the provenance information, and the accuracy of this information cannot be readily verified.

Provenance, however, must be considered in a broader context. To ensure quality, it is not sufficient to know that a particular data item was inferred computationally; it is also important to identify which data points contributed to the inference. Such information can be used, e.g. to invalidate the inferred data if part of the data on which the inference was based is later found to be incorrect or abnormal, or if our understanding of biology has evolved. Determining the influence of a particular data point on the output of an inference engine is complex and remains an active area of research in machine learning (Hammoudeh and Lowd 2024).

Furthermore, public data sets are often the basis for major studies underlying new biological discoveries. Knowing which study leveraged which data set (or parts thereof), and which version of the data was used in the analysis, is important for reproducibility, and also for allowing findings to be updated or invalidated if the original data are corrected or found to be erroneous. Such data sources are also often used in benchmarking new methods, and failure to account for dependencies between data sets can undermine sound statistical cross-validation practice in making these evaluations. We are not aware of any practical attempts to capture provenance at this broader level.

## 7 Education is critical

The points we have raised so far are generally well appreciated by experts in computational biology and machine learning (De Crécy-lagard *et al.* 2022), but are often overlooked by the vast majority of scientists and engineers engaged in data-intensive computational biology research and by end-user life scientists. This knowledge gap is due to the broad diversity of the computational biology field, which encompasses scientists with varied educational backgrounds across many disciplines (Işık *et al.* 2023). Increasingly, modern biological research results in the generation of large volumes of data, and there is a growing need for even primarily experimental scientists to use sophisticated tools for data analysis. Efforts to define the core computational biology competencies needed from all life scientists are already underway (Brooksbank *et al.* 2024). New educational programs that teach practitioners, regardless of discipline or academic background, the fundamentals of data handling and information and error propagation throughout analytic pipelines must be developed. Developing such programs will require recognition across the life sciences that such training is essential, even if providing it faces significant institutional obstacles (Williams *et al.* 2019). Conversely, the growing need for data analytics in the life sciences means that increasingly biological data analyses are performed by data scientists without formal biological training, who themselves can benefit from new educational programs (Attwood *et al.* 2019, Via *et al.* 2019). Such programs, whether targeted at life scientists or data scientists, will require pedagogical innovations to personalize the curriculum and teaching strategies to the diverse backgrounds of the trainees (Tractenberg *et al.* 2019). Recent advances in AI and their potential application in biology have created new courses introducing machine learning and AI to biologists. We encourage those who develop such courses to also focus on the issues surrounding the data on which such tools are applied (data engineering) in addition to the more common attention given to the algorithmic and statistical underpinnings of machine learning tools. It is also essential to discuss the nuance associated with the labels assigned to biological entities, such as taxonomic labels, GO terms, etc. As we have already discussed, such labels do not represent an inherent biological truth; instead, they are a representation of the current understanding of biology at the time when the labels were created.

## 8 Best practices for data use and re-use

To address rigor and reproducibility, standards and best practices have been developed in our field, such as various minimum information standards for sequences (Brazma *et al.* 2001, Taylor *et al.* 2008, Yilmaz *et al.* 2011, Rustici *et al.* 2021) or the FAIR (Findable, Accessible, Interoperable, and Reusable) principles for data management (Wilkinson *et al.* 2016). These address issues such as required data descriptions, formats, and access. BioDBCore (Gaudet *et al.* 2011), an early attempt to encourage consistency and interoperability between resources and to promote the use of semantic and syntactic standards, was undertaken prior to the current AI boom and it is time to update this approach. An argument has also been made for a stronger integration of databases and publications to both facilitate the computational interpretation of biological data and to enable data validation (Bourne 2005, Attwood *et al.* 2009). Furthermore, some publications have proposed best practices for utilizing machine learning in computational biology (Chicco 2017, Greener *et al.* 2022, Lee *et al.* 2022), and for making data sets discoverable (Contaxis *et al.* 2022) and reusable (Conte *et al.* 2024).

None of these proposed best practices adequately address the issues that arise from the re-use of computationally-generated data. Such issues include: (i) adequately documenting the computational methods used to generate the data in publications and databases; (ii) recording the method of data generation sufficiently to determine whether the data are suitable for particular subsequent analyses; and (iii) providing standardized and quantifiable confidence measures. Developing recommended practices will require broader community discussion, consensus, and involvement.

## 9 Discussion and recommendations

We have highlighted several issues related to computationally generated data in biological databases and their use in computational analyses. Problems may arise from computational analysis of experimental data, which later prove to be in error as fields evolve, or from computation alone, which leads to propagation of errors, mis-annotation being a significant example. We recognize that these problems are not new, and the consortia that maintain public databases and knowledgebases already largely embrace the recommendations we make below. However, the issues surrounding data quality and error propagation need to be better understood by the broad community of users of data, biologists and computational scientists alike. This need is particularly relevant in light of modern AI’s ability to generate vast numbers of annotations and models and its need for vast amounts of data for training. Without a broader recognition of the issues outlined in this perspective and immediate action on them, databases and knowledgebases will be increasingly corrupted. The authors of this article, Fellows of the International Society for Computational Biology (ISCB), make the following recommendations, intended to address these issues, to funders and to the broadly-defined computational biology community. We commit to working with the ISCB and allied research communities representing developers of biological databases and knowledgebases to implement these recommendations. We must move the broader community forward to benefit from more accurate data that drives biological discovery.

**Recommendation 1—Develop an explicit education directive.** Such a directive should include hands-on training in the practical use of machine learning and computational analytics, and the development of training material that highlights common pitfalls that derive from data artifacts and the propagation of errors across tools and databases. Program materials should be open and widely disseminated. We recommend that funders allocate resources appropriately to meet the needs of these programs. We further recommend that the Education Committee of the ISCB, as well as other organizations collaborating under the umbrella of the Global Organization for Bioinformatics Learning, Education, and Training (GOBLET) (Attwood *et al.* 2015), make nurturing the development of these education modules a priority. Educating users on the proper use of data is not optional.

**Recommendation 2—Support the study of error propagation as bona fide scholarship.** We are computational biologists undertaking experimental and theoretical research. Let us apply some of that knowledge and skill to develop a computational biology-specific evidence base upon which best practices can be developed to minimize errors and error propagation. This research should also focus on the related issue of discovering missing data/knowledge and enabling better approaches for detecting errors. Let us develop quantitative measures of the error present in major databases. Let us have sessions at ISCB meetings that present progress in this endeavor.

**Recommendation 3—Develop improved data provenance mechanisms that explicitly account for computationally generated data.** Funders insist on data management plans. What use are those data if incorrect and not appropriately labeled as to their derivation and other quality indicators? Funders should require data management plans that account for computationally generated data, and that ensure rich provenance data are provided (e.g. including confidence estimates in addition to software/data versions), and they should fund resources to support data stewardship.

**Recommendation 4—Develop mechanisms to support the stewardship of public biological data resources.** Biological data and knowledge are not static; even experimental evidence may be reinterpreted as our understanding of biology evolves. Computation can propagate, amplify, or “bake in” historical artifacts no longer compatible with modern knowledge. Funders should support efforts to update and correct the information in the databases and knowledgebases they fund, and ISCB should support the dissemination of and education on this work and its progress.

**Recommendation 5—Hold the computational biology community accountable for the stewardship of the biological data ecosystem.** Advancing the recommendations we make here will require intentional and sustained effort. We propose developing quantitative measures that can be used to assess our progress. ISCB should convene regular meetings during which our community can develop appropriate quantitative measures, and use them to take stock of the field’s current state, identify current trends and emerging challenges, and prioritize future actions aimed at enhancing the quality and utility of biological data sets.

Data are available globally and are subject to different governance, access restrictions, etc., yet the problems discussed here transcend national and domain boundaries. ISCB is an international society and needs to consider this variance and encourage sharing knowledge on addressing these problems. Biologists were pioneers in using digital data, but digital data now drive every academic field—and the problems described here are not unique to biology. We champion an interdisciplinary approach to the issues highlighted in this article.

## Data Availability

No data is associated with this manuscript since it is a perspective.
